# A Lightweight Forward-Looking Sonar Sensing Framework for Embedded Target Detection in Resource-Constrained Underwater Systems

**DOI:** 10.3390/s26103133

**Published:** 2026-05-15

**Authors:** Hong Peng, Chaolin Yang, Chen He, Wei Ye, Renyou Yang

**Affiliations:** Southern Marine Science and Engineering Guangdong Laboratory (Zhanjiang), Zhanjiang 524002, China; penghong@zjblab.com (H.P.); yangchaolin@zjblab.com (C.Y.); hechen@zjblab.com (C.H.); yewei@zjblab.com (W.Y.)

**Keywords:** forward-looking sonar sensing, embedded sonar perception, lightweight target detection, resource-constrained sensing systems, knowledge distillation, autonomous underwater vehicles

## Abstract

Forward-looking sonar (FLS) is an important sensing modality for autonomous underwater vehicles and other marine robotic systems operating in turbid, low-visibility, and acoustically cluttered environments. Reliable target detection in FLS imagery remains challenging because target echoes are often weak, compact targets can be obscured by background clutter, and embedded processors impose strict limits on model size, latency, and computation. To address these issues, this study presents a lightweight FLS sensing framework for embedded target detection in resource-constrained underwater systems. The framework combines a compact detection architecture, difficulty-aware supervision, and teacher–student knowledge transfer. Specifically, FPN-Mix is developed as a lightweight backbone with a Conv-Mix module to improve contextual aggregation under limited computational budgets. A target-aware dynamic weighting loss is introduced to increase the supervision weight of difficult acoustic samples associated with weak echoes, ambiguous boundaries, and clutter interference. A multi-level knowledge distillation strategy is then adopted to transfer feature-level and prediction-level knowledge from an enhanced teacher model to the compact student detector. Experiments on the public UATD benchmark and the independently collected Zhanjiang Bay No.1 field dataset show that the proposed method achieves a favorable balance between detection accuracy and efficiency and remains competitive in a real marine aquaculture environment. The proposed model contains only 2.83 M parameters and requires 6.68 GFLOPs. After ONNX export and TensorRT FP16 acceleration, the model reaches 72.23 frames per second (FPS) on an NVIDIA Jetson Orin NX platform, supporting its practical use in embedded FLS sensing systems.

## 1. Introduction

Forward-looking sonar (FLS) is an important sensing modality for autonomous underwater vehicles (AUVs) and other marine robotic platforms operating in turbid, low-visibility, and acoustically cluttered environments. Unlike optical cameras, FLS can provide stable scene perception when light attenuation, suspended particles, and water turbidity severely degrade visual sensing. This makes FLS highly valuable for underwater inspection, obstacle avoidance, seabed exploration, and autonomous navigation [1,2,3,4]. In these scenarios, target detection is not merely an isolated algorithmic task; rather, it is a core perception function within a complete sonar sensing system whose practical utility depends on both detection quality and deployability.

Despite its sensing advantages, FLS imagery remains substantially more difficult to interpret than optical imagery. Acoustic targets often appear with weak signatures, blurred contours, sparse texture, heavy reverberation, and strong background clutter. Targets occupying limited image areas or presenting weak acoustic responses are especially vulnerable to missed detection, while background structures can easily trigger false alarms. Early sonar detection methods mainly relied on handcrafted features and rule-based processing. Representative examples include symbolic pattern analysis [5], texture-based discrimination of man-made objects [6], edge-guided segmentation [7], and multi-algorithm fusion schemes [8,9]. Although effective in constrained settings, these methods depend heavily on manually designed priors and empirical thresholds, which limits robustness across dynamic underwater environments.

Recent progress in deep learning has substantially advanced target perception in optical imagery, with influential detector families such as R-CNN [10,11,12,13], YOLO [14,15], and transformer-based detectors [16,17]. Motivated by these successes, researchers have begun transferring deep models to sonar imagery. For example, Faster R-CNN has been adapted for wreckage detection in side-scan sonar (SSS), and comparative studies have confirmed the feasibility of convolutional neural network (CNN)-based sonar perception [18]. However, directly applying optical-image detectors to sonar data is often insufficient. Compared with optical images, FLS data exhibit lower contrast, stronger clutter, weaker target boundaries, and more severe noise interference, all of which reduce the reliability of off-the-shelf detection architectures.

A second key challenge lies in the strict resource constraints of practical underwater sensing systems. In onboard AUV deployment, perception modules must simultaneously satisfy latency, memory, and power requirements. Large models may improve benchmark accuracy but are often unsuitable for real-time inference on embedded processors. Consequently, the objective is not only to improve detection accuracy, but also to design a framework that remains lightweight and deployable.

This requirement is especially critical for FLS. Compared with SSS, FLS provides wider field-of-view sensing, greater angular flexibility, and more convenient integration with mobile robotic platforms, but typically at the cost of lower spatial resolution and weaker target details. Existing sonar studies still focus predominantly on accuracy-oriented detector design or on SSS imagery, whereas fewer works explicitly address the system-level need for lightweight FLS perception with validated embedded deployment. This gap is substantial because a practically useful FLS perception framework should demonstrate not only improved inference quality but also credible feasibility on real embedded hardware.

This work treats FLS target detection as an embedded sensing problem rather than a desktop-only detection benchmark. The proposed framework is designed for practical sonar perception, where detection accuracy and computational cost must be considered together. Feature extraction, supervision design, and teacher–student transfer are jointly considered to maintain detection performance while reducing the burden of deployment.

The main contributions of this work are summarized as follows:

1. A lightweight FLS sensing framework is developed based on an FPN-Mix backbone and a Conv-Mix module. This design improves contextual modeling in cluttered FLS imagery while keeping the detector compact enough for embedded deployment.
2. To improve training on weak and ambiguous acoustic targets, a target-aware dynamic weighting loss is introduced. This loss increases the contribution of difficult samples caused by weak echoes, unclear boundaries, and background clutter without adding inference cost.
3. A multi-level teacher–student distillation strategy is designed to combine attention-guided feature transfer, task-level prediction supervision, and FPN-level distillation. This strategy enables the compact student detector to learn more discriminative representations from a stronger teacher model.
4. The proposed framework is evaluated through benchmark testing, field validation, and embedded deployment. In addition to experiments on the public UATD benchmark, the method is further validated on the independently collected Zhanjiang Bay No.1 field dataset to examine its generalization ability in a real marine aquaculture environment. The model achieves a competitive accuracy–efficiency trade-off with 2.83 M parameters and 6.68 GFLOPs, and reaches 72.23 FPS after TensorRT FP16 deployment on an NVIDIA Jetson Orin NX platform.

The remainder of the paper first reviews related studies on sonar target detection and KD. The proposed lightweight FLS sensing framework is then described in detail, followed by quantitative comparisons, ablation studies, generalization evaluation, qualitative visualization, and embedded deployment results. The paper concludes with a summary of the main findings and possible future directions.

## 2. Related Work

Efficient FLS perception requires a careful balance between representation quality and computational cost, especially when the detector is intended to function as an embedded sensing module rather than a desktop-only benchmark model. This section reviews two groups of studies most relevant to this work: target detection in sonar imagery and KD for efficient detection.

### 2.1. Target Detection in Underwater Sonar Imagery

With the rapid progress of optical object detection [11,12,13,14,19,20,21,22,23,24,25,26,27,28,29,30,31,32,33,34,35,36], deep models have been increasingly transferred to sonar imagery. Early studies mainly adapted optical detectors to SSS data through multi-scale feature fusion, transfer learning, and task-specific refinement. Representative examples include improved YOLOv3 for small SSS datasets [37], Mask R-CNN variants for sonar target localization [38], lightweight residual backbones for parameter reduction [39], and noise-aware detection frameworks such as NFD-YOLO [40]. Other efforts further improved SSS detection through shadow-aware modeling [41], multi-scale attention mechanisms [42], anchor assignment refinement [43], graph-based channel attention [44], and unsupervised shadow detection [45].

However, most of these studies focus on SSS rather than FLS. Compared with SSS, FLS provides wider coverage and greater deployment flexibility, but usually suffers from lower spatial resolution, weaker target details, and heavier clutter interference. As a result, methods developed for SSS or optical imagery cannot be directly transferred to FLS perception without adaptation to acoustic imaging characteristics [46,47,48].

Recent FLS-oriented studies have explored hybrid CNN–Transformer architectures and lightweight detection models [49,50,51]. These methods have improved detection performance, but maintaining a reliable balance between accuracy and efficiency remains difficult under onboard deployment constraints. In addition, knowledge distillation tailored to FLS imagery remains relatively underexplored. These limitations motivate the development of a lightweight FLS detection framework that preserves detection accuracy while remaining deployable on resource-constrained underwater platforms.

### 2.2. Knowledge Distillation

As discussed above, onboard deep models must balance detection accuracy and computational efficiency. Although large models usually achieve stronger representation capability and better detection performance, they also require substantially higher computational resources [52,53]. To address this issue, model compression techniques such as pruning have been widely studied. Knowledge distillation, originally proposed for image classification [54], transfers knowledge from a large teacher model to a lightweight student model and has become an effective paradigm for efficient model design.

Subsequent studies extended KD to object detection. Chen et al. [55] demonstrated its effectiveness for compressing SSD detectors by introducing a hint-based loss on intermediate features. Wei et al. [56] further enabled student detectors to learn from both final predictions and intermediate representations, improving performance in compact models. Zhang et al. [57] applied KD to Faster R-CNN and achieved improved efficiency without sacrificing detection accuracy. Yang et al. [58] further applied a knowledge-distillation-based strategy to conveyor belt defect detection, demonstrating the applicability of KD in industrial defect detection scenarios. Zheng et al. [59] proposed localization distillation to transfer bounding-box localization knowledge, while Wang et al. [60] introduced CrossKD, which transfers intermediate features from the student’s detection head to the teacher’s head to alleviate the conflict between ground-truth supervision and teacher guidance.

Despite these advances, knowledge distillation has been used only sparingly for underwater sonar target detection. Since compact detectors are needed for AUVs and other embedded underwater platforms, sonar-oriented distillation remains a useful direction for efficient FLS perception.

## 3. Proposed Method

**Figure 1:**
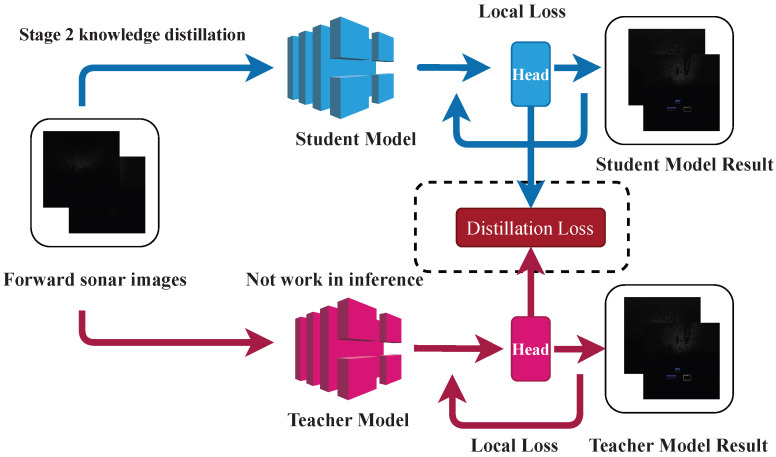
Overall framework of the proposed KD method. The same FLS image is fed into both the teacher and student networks. Multi-scale features are extracted and passed to the corresponding detection heads for task-loss computation, while intermediate and output representations are aligned through distillation losses to optimize the student network.

As shown in Figure 1, the proposed framework follows a teacher–student detection pipeline for embedded FLS sensing. The input FLS image is first processed by the backbone to extract hierarchical features, which are then fused by the feature pyramid network (FPN) and passed to decoupled detection heads for classification and localization. During training, the student detector is supervised by both the standard detection loss and the distillation losses from the teacher model.

The framework is built on a one-stage detector and is designed to improve FLS feature representation while keeping the student model compact for embedded deployment. FPN-Mix is used to strengthen contextual feature extraction under weak and cluttered acoustic responses, TADW adjusts the learning emphasis on difficult samples, and multi-level teacher–student distillation helps compensate for the representation loss caused by lightweight model design.

### 3.1. Ultra-Lightweight Network Architecture

#### 3.1.1. Multi-Scale Feature Integration Mixture Network

FLS targets often appear with scale-varying acoustic responses and incomplete structural cues across different feature levels. A single local convolution branch can preserve fine details, but it is less effective at integrating multi-scale acoustic context when weak target responses are mixed with background reverberation. To improve feature aggregation under this condition, an FPN-Mix backbone equipped with the Conv-Mix module is introduced. The goal is to enhance multi-scale contextual representation while keeping the feature extraction process lightweight for embedded deployment.

**Figure 2:**
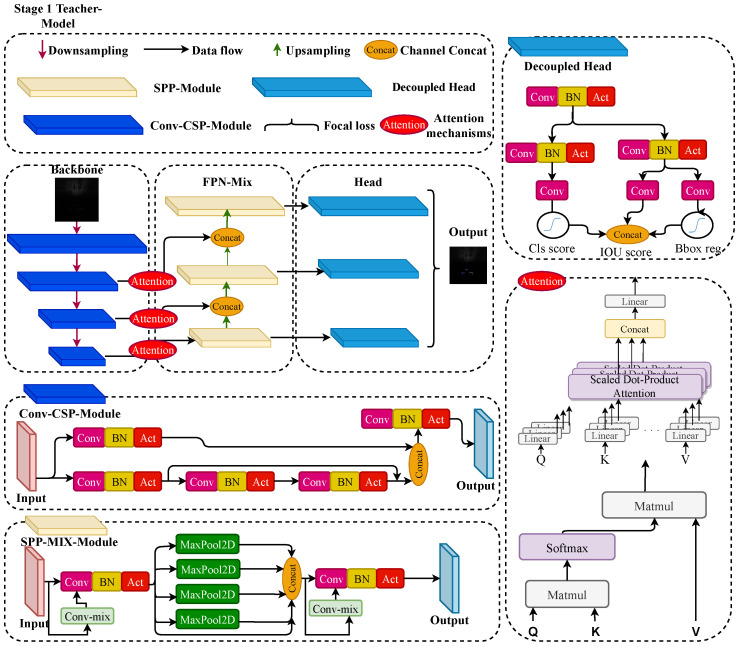
Architecture of the teacher model. The input FLS image is first processed by the backbone for feature extraction. In the teacher branch, attention modules are inserted before the neck for enhanced feature refinement, whereas the lightweight student branch removes these modules to satisfy deployment constraints.

As illustrated in Figure 2, the FPN-Mix block uses a parallel structure that combines a baseline convolution branch and a Conv-Mix branch. The baseline branch preserves local spatial responses, whereas the Conv-Mix branch introduces region-aware contextual information. Their fusion allows the network to retain local acoustic details while improving the representation of target-related structures across different scales.

Let F denote the input feature map to the FPN-Mix block. The two parallel branches are first fused as  

(1) $$Z=f_{\mathrm{conv}-\mathrm{mix}}\left(F\right)⊕f_{\mathrm{base}}\left(F\right),$$

where ⊕ denotes element-wise addition, $f_{\mathrm{conv}-\mathrm{mix}}\left(\cdot \right)$ denotes the Conv-Mix branch, and $f_{\mathrm{base}}\left(\cdot \right)$ denotes the baseline convolution branch.

To aggregate multi-scale information, the fused feature Z is pooled at four scales, resized to a common spatial size, and concatenated along the channel dimension:

(2) $$Y={\mathrm{Cat}}_{i=1}^{4}\left[\mathrm{Resize}\;\left(f_{\mathrm{pool}}^{\left(i\right)}\left(Z\right)\right)\right],$$

where $f_{\mathrm{pool}}^{\left(i\right)}\left(\cdot \right)$ denotes the pooling operation at the *i*-th scale, Resize(·) denotes spatial alignment, and Cat(·) denotes channel-wise concatenation.

The aggregated multi-scale representation is then refined by another branch-fusion operation:

(3) $$F_{\mathrm{mix}}=f_{\mathrm{conv}-\mathrm{mix}}\left(Y\right)⊕f_{\mathrm{base}}\left(Y\right).$$

The fused features are finally propagated to three pyramid output levels with progressively reduced spatial resolutions, where *H* and *W* denote the input spatial dimensions and $C_{i}$ denotes the channel dimension of the *i*-th output level.

#### 3.1.2. Conv-Mix Module

In FLS images, target-related acoustic responses are often distributed over small acoustic regions and are affected by fan-shaped imaging geometry, uneven echo intensity, and surrounding reverberation. A standard convolutional branch mainly aggregates information within fixed local neighborhoods. Such local aggregation is less effective at relating a response to its surrounding acoustic context, which can make the detector sensitive to isolated high-intensity background responses and less capable of preserving weak but structurally meaningful target cues.

**Figure 3:**
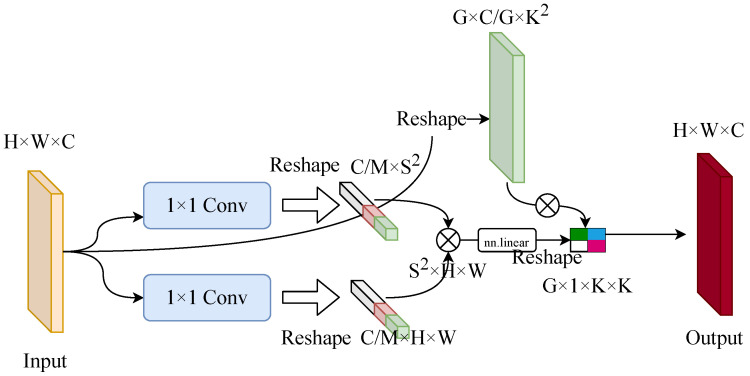
Architecture of the Conv-Mix module.

To address this issue, Conv-Mix introduces an efficient token-to-region interaction mechanism. Instead of performing dense global self-attention, the module first summarizes the feature map into a set of pooled regional descriptors and then allows spatial tokens to selectively interact with these descriptors. This design interprets local responses with reference to regional acoustic patterns while keeping the computation suitable for a lightweight embedded detector. And the architecture of the Conv-Mix module is illustrated in Figure 3.

First, the spatial token representation, regional key representation, and regional value representation are generated as

(4) $$\begin{array}{cc} P & =\mathrm{reshape}(W_{p}X),\\ C & =\mathrm{reshape}\left(W_{c}\mathrm{Pool}\left(X\right)\right),\\ V & =\mathrm{reshape}\left(W_{v}\mathrm{Pool}\left(X\right)\right), \end{array}$$

where $X\in R^{C\times H\times W}$ denotes the input feature map, $W_{p}$, $W_{c}$, and $W_{v}$ are 1×1 convolutional projections, Pool(·) denotes adaptive average pooling to regional summaries, and reshape(·) flattens the spatial dimensions. To reduce computation, the channel dimension is divided into *M* groups, and token-to-region interaction is computed independently within each group.

For the *m*-th group, the contextual response is obtained by

(5) $$O^{\left(m\right)}=V^{\left(m\right)}{\left[\mathrm{Softmax}\;\left({\left(P^{\left(m\right)}\right)}^{⊤}C^{\left(m\right)}\right)\right]}^{⊤},\;m=1,\ldots ,M.$$

Here, ${\left(P^{\left(m\right)}\right)}^{⊤}C^{\left(m\right)}$ measures the affinity between spatial tokens and regional summaries, while the row-wise softmax produces adaptive mixing weights. Through this operation, each spatial token is updated according to its affinity with pooled regional descriptors, allowing the module to suppress isolated clutter responses and retain target-related acoustic structures more effectively.

The group-wise outputs are then concatenated, reshaped back to the spatial domain, and projected to form the final Conv-Mix output:

(6) $$f_{\mathrm{conv}-\mathrm{mix}}\left(X\right)=W_{o}\;\mathrm{reshape}\;\left(\mathrm{Cat}\left(O^{\left(1\right)},O^{\left(2\right)},\ldots ,O^{\left(M\right)}\right)\right),$$

where $W_{o}$ denotes the output projection and Cat(·) denotes channel-wise concatenation. The resulting Conv-Mix output combines local spatial responses with region-level acoustic cues, providing a compact alternative to full attention for embedded FLS perception.

### 3.2. Target-Aware Dynamic Weighting Loss

In FLS detection, training samples often exhibit substantial differences in optimization difficulty. Many background regions are easy to classify, whereas foreground targets and target-like clutter tend to produce low-confidence predictions during training. If all samples are weighted uniformly, abundant easy background responses can dominate the loss, reducing the effective supervision for hard foreground regions and confusing background patterns. To address this issue, TADW is introduced to adaptively increase the supervision weight of difficult acoustic samples according to the evolving optimization state.

#### TADWLoss Formulation

Let *p* denote the predicted probability for a binary target label *y*. The standard compact notation $p_{t}$ is defined as

(7) $$p_{t}=\left\{\begin{array}{cc} p, & y=1,\\ 1-p, & y=0. \end{array}\right.$$

A lower value of $p_{t}$ indicates a more difficult sample. To make the weighting process adaptive to the current training state, a smoothed confidence estimate is introduced as

(8) $${\hat{p}}_{t}=\beta \left(\frac{1}{t}\sum_{i=1}^{t}p_{i}\right)+\left(1-\beta \right)p_{t},$$

where β=0.05 controls the balance between historical and current prediction confidence. Based on $p_{t}$ and ${\hat{p}}_{t}$, two dynamic factors are defined as $\delta_{t}=-\ln\left(p_{t}\right)$ and $\omega_{t}=-\ln\left({\hat{p}}_{t}\right)$, where $\delta_{t}$ reflects sample-specific difficulty and $\omega_{t}$ describes the smoothed training state.

The final TADW loss is formulated as  

(9) $$L_{\mathrm{TADW}}=\left\{\begin{array}{cc} -{\left(\alpha -p_{t}\right)}^{\delta_{t}}\log\left(p_{t}\right), & p_{t}<\theta ,\\ -{\left(1-p_{t}\right)}^{\omega_{t}}\log\left(p_{t}\right), & p_{t}\geq \theta , \end{array}\right.$$

where θ is set to 0.5 by default, and α controls the reweighting strength for difficult samples. In this way, TADW adaptively increases the supervision weight of difficult acoustic samples without introducing additional inference-time complexity.

Compared with standard focal loss, which uses a fixed modulation factor ${\left(1-p_{t}\right)}^{\gamma }$ to down-weight easy samples, TADW introduces the dynamic factors $\delta_{t}$ and $\omega_{t}$ to adjust the weighting strength according to the current prediction confidence and its smoothed historical estimate. Therefore, the sample weighting in TADW can vary with both sample difficulty and the evolving training state. This adaptive behavior is beneficial for FLS imagery, where target-like clutter, weak acoustic responses, and foreground–background ambiguity often lead to large variations in prediction confidence during training.

### 3.3. Multi-Level Knowledge Distillation Framework

Lightweight detector design is necessary for embedded deployment, but aggressive model compression can reduce the representation capacity of the student model. This is especially problematic in FLS imagery, where targets often have weak responses and are easily confused with clutter. To reduce this loss of discrimination, a multi-level teacher–student distillation framework is introduced that transfers feature-level and prediction-level knowledge from a stronger teacher network to the lightweight student detector.

Given a teacher network $f_{T}\left(x\right)$ and a student network $f_{S}\left(x\right)$, an asymmetric distillation design is used. Attention modules are retained in the teacher branch to improve feature refinement during training, while the student branch removes these modules to keep inference efficient. With this design, the teacher can provide stronger training guidance, whereas the deployed student does not incur additional inference cost.

**Figure 4:**
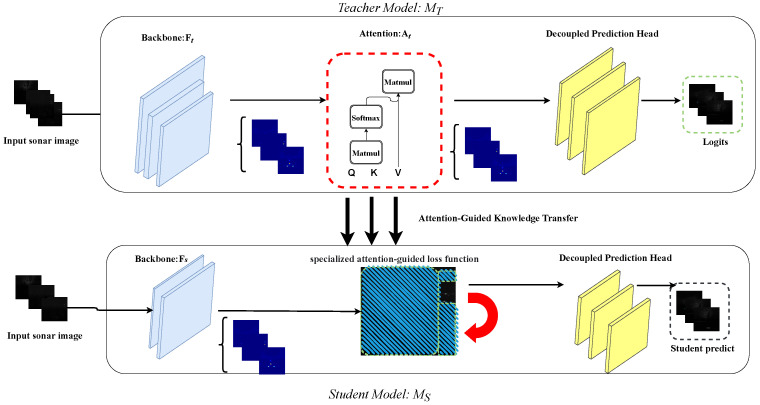
Teacher–student attention transfer strategy. Attention modules are retained in the teacher network to enhance feature refinement during training, whereas the student network removes these modules for efficient deployment. Distillation losses are used to transfer the teacher’s attention-enhanced representations to the lightweight student.

As illustrated in Figure 4, the teacher branch provides attention-enhanced feature representations, while the lightweight student learns from the teacher through both attention-guided feature distillation and prediction-level distillation over classification, localization, and objectness branches.

#### 3.3.1. Attention-Guided Feature Distillation

To transfer the teacher’s attention-enhanced representations to the student, the feature distillation loss is defined as

(10) $$L_{\mathrm{feat}}={∥A_{t}⊙f_{T}\left(x\right)-f_{S}\left(x\right)∥}_{2}^{2},$$

where $A_{t}$ denotes the attention map of the teacher model, $f_{T}\left(x\right)$ and $f_{S}\left(x\right)$ denote the teacher and student feature representations, respectively, and ⊙ represents element-wise multiplication. This term encourages the student to align with the attention-refined features learned by the teacher.

#### 3.3.2. Prediction-Level Knowledge Alignment

In addition to feature alignment, the teacher’s prediction knowledge is further distilled from the classification, bounding-box regression, and objectness branches.

For classification distillation, the Kullback–Leibler (KL) divergence between the softened teacher and student predictions is minimized:

(11) $$L_{\mathrm{KD}-\mathrm{cls}}=\sum_{k=1}^{K}p_{k}^{T}\left(x\right)\log\frac{p_{k}^{T}\left(x\right)}{p_{k}^{S}\left(x\right)},$$

where *K* is the number of categories. The teacher probability is computed with a temperature parameter τ as

(12) $$p^{T}\left(x\right)=\mathrm{softmax}\left(z^{T}\left(x\right),\tau \right)=\frac{\exp\left(z^{T}\left(x\right)/\tau \right)}{\sum_{j=1}^{K}\exp\left(z_{j}^{T}\left(x\right)/\tau \right)},$$

where $z^{T}\left(x\right)$ denotes the logits produced by the teacher network for sample *x*. The student probability $p^{S}\left(x\right)$ is defined analogously.

For bounding-box regression distillation, the mean squared error is used to align the teacher and student box predictions:

(13) $$L_{\mathrm{KD}-\mathrm{bbox}}=\frac{1}{N}\sum_{i=1}^{N}{\left({\mathrm{Reg}}_{\mathrm{output}}^{T}-{\mathrm{Reg}}_{\mathrm{output}}^{S}\right)}^{2},$$

where *N* is the number of bounding-box predictions.

For objectness distillation, the binary cross-entropy loss is adopted:

(14) $$\begin{array}{cc} L_{\mathrm{KD}-\mathrm{obj}} & =-\frac{1}{N}\sum_{i=1}^{N}[{\mathrm{Obj}}_{t}^{\left(i\right)}\log\left({\mathrm{Obj}}_{s}^{\left(i\right)}\right)\\ \; & \;+\left(1-{\mathrm{Obj}}_{t}^{\left(i\right)}\right)\log\left(1-{\mathrm{Obj}}_{s}^{\left(i\right)}\right)], \end{array}$$

where ${\mathrm{Obj}}_{s}={\left\{{\mathrm{Obj}}_{s}^{\left(i\right)}\right\}}_{i=1}^{N}$ and ${\mathrm{Obj}}_{t}={\left\{{\mathrm{Obj}}_{t}^{\left(i\right)}\right\}}_{i=1}^{N}$ denote the student and teacher objectness predictions, respectively.

#### 3.3.3. Multi-Level Distillation Across FPN Features

Because FLS imagery often contains weak and scale-varying target responses embedded in cluttered backgrounds, multi-level distillation is applied across the FPN outputs to enhance the student’s representation capability at different semantic scales. Specifically, the prediction-level distillation losses for classification, bounding-box regression, and objectness are averaged over the three FPN levels, allowing the student to absorb discriminative teacher knowledge from coarse semantic cues to finer localization details.

#### 3.3.4. Overall Distillation Objective

Based on the above definitions, the soft distillation objective is written as

(15) $$L_{\mathrm{KD}-\mathrm{soft}}=\lambda_{\mathrm{feat}}L_{\mathrm{feat}}+\lambda_{\mathrm{cls}}L_{\mathrm{KD}-\mathrm{cls}}^{\mathrm{multi}}+\lambda_{\mathrm{bbox}}L_{\mathrm{KD}-\mathrm{bbox}}^{\mathrm{multi}}+\lambda_{\mathrm{obj}}L_{\mathrm{KD}-\mathrm{obj}}^{\mathrm{multi}}$$

where $\lambda_{\mathrm{feat}}$, $\lambda_{\mathrm{cls}}$, $\lambda_{\mathrm{bbox}}$, and $\lambda_{\mathrm{obj}}$ are balancing coefficients.

The final training objective is then formulated as

(16) $$L_{\mathrm{total}}=L_{\mathrm{hard}}+L_{\mathrm{KD}-\mathrm{soft}}$$

where $L_{\mathrm{hard}}$ denotes the standard detection loss of the student network. In this way, the student is optimized not only by ground-truth supervision but also by multi-level guidance from the teacher, which improves feature representation and detection robustness in challenging FLS scenes.

## 4. Experimental Results and Analysis

### 4.1. Dataset

**Figure 5:**
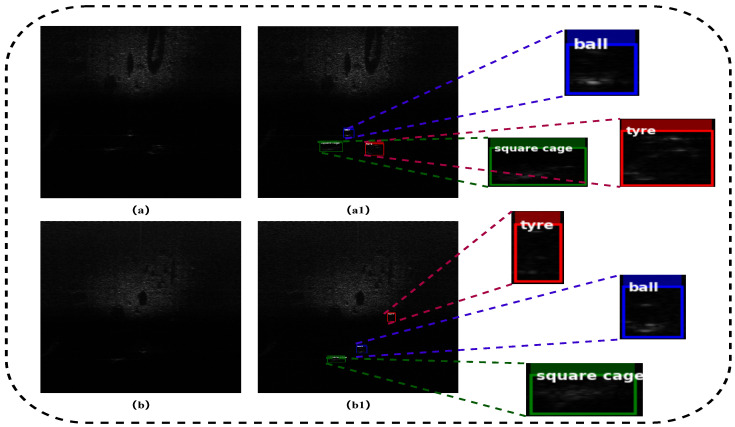
Representative examples from the UATD dataset: (**a**) raw FLS image from UATD-Test-1; (**a1**) manual annotation corresponding to (**a**); (**b**) another raw FLS image from the UATD-Test-2; and (**b1**) manual annotation corresponding to (**b**).

**Figure 6:**
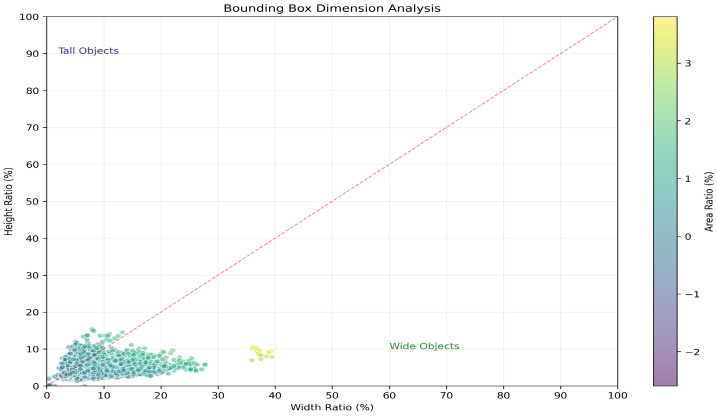
Distribution of target sizes in the UATD dataset. Most annotated targets occupy only a limited fraction of the full image area, highlighting the difficulty of detecting relatively compact target regions in FLS imagery.

The proposed framework is evaluated on the 2022 Underwater Acoustic Target Detection (UATD) dataset, a public benchmark for FLS target detection released by Xie et al. [61]. The dataset provides a standardized fan-to-matrix conversion and contains more than 9000 images acquired with a Tritech Gemini 1200ik sonar (Tritech International Limited, Westhill, Aberdeenshire, United Kingdom), covering 10 target categories with raw sonar data and corresponding annotations. The original release defines three predefined subsets: UATD_Training, UATD_Test_1, and UATD_Test_2, including 7600 training images and two independent test subsets of 800 images each. This official split is followed without additional random re-splitting or redistribution: UATD_Training is used for model training, while UATD_Test_1 and UATD_Test_2 are used for independent evaluation. Figure 5 shows representative examples from the dataset, and Figure 6 shows the target-size distribution, where most bounding boxes occupy only about 10% of the full image area, reflecting the difficulty of detecting targets that occupy limited regions in FLS imagery.

The standard COCO AP_small_ metric is not used as the primary scale-level metric in this study because the official UATD test subsets contain very few instances satisfying the COCO-defined small-object criterion of an area smaller than 32×32 pixels. Such a limited number of samples makes AP_small_ statistically unstable and sensitive to a few detections. Therefore, a dataset-specific compact-target evaluation is introduced according to the relative bounding-box area in the original FLS image.

For each annotated target, the relative area is defined as (w×h)/(W×H), where *w* and *h* denote the width and height of the bounding box, and *W* and *H* denote the width and height of the original sonar image. According to the target-size distribution shown in Figure 6, targets with a relative area smaller than 0.3% are selected as compact targets. The corresponding compact-target AP is reported as AP_compact_. This evaluation better reflects the scale characteristics of FLS imagery, where detection difficulty is closely related to the fraction of the sonar image occupied by the acoustic response rather than only to the absolute pixel area.

### 4.2. Experimental Settings

All comparative experiments were conducted on a desktop platform using PyTorch 2.5.0+cu124 and MMDetection with an NVIDIA RTX 3060 GPU. The batch size was set to 4, and the models were optimized using stochastic gradient descent (SGD) with a momentum of 0.9. The initial learning rate was set to 0.0003125 and updated using a cosine annealing schedule. Unless otherwise stated, the hyperparameter α in TADW was fixed at 1.5 in the main comparison experiments, while a separate sensitivity study was conducted to examine different α values. All models were trained for 12 epochs.

All detectors followed the same dataset split, input resolution, batch size, training length, and evaluation metrics. The 12-epoch setting follows the standard MMDetection 1× training schedule and provides a common baseline for detector comparison under the adopted dataset scale and batch size. Classical detectors, including YOLOv5-S, YOLOv7-Tiny, EfficientDet-Lite-D1, NanoDet, RetinaNet, PAA, VFNet, TOOD, and Sparse R-CNN, were implemented using the MMDetection framework and its corresponding configuration files. Recent state-of-the-art detectors, including RF-DETR, Salience-DETR, FFCA-YOLO, and Hyper-YOLO-N, were evaluated using their publicly released official implementations provided by the original authors, with dataset paths, class numbers, input resolution, batch size, training epochs, and evaluation metrics adjusted to match the same experimental protocol.

For quantitative evaluation, mAP@50, mAP@50:95, parameter count, GFLOPs, and inference speed are reported. Desktop-side benchmarking results, including parameter count, GFLOPs, and FPS, were obtained under the same software environment and input setting, and are used for fair comparison among different detectors. The corresponding desktop-side complexity and inference-speed results are reported later in the desktop-side complexity comparison.

To evaluate embedded deployment, the proposed model was further exported to ONNX and accelerated with TensorRT FP16 inference on an NVIDIA Jetson Orin NX platform. The embedded results are reported separately in Section 4.7 and are used to verify the practical edge-side deployment capability of the proposed method rather than to replace the desktop-side comparison protocol.

All detectors were trained under a unified data preprocessing pipeline. For each sample, the input image and the corresponding bounding-box annotations were loaded, and invalid annotations were filtered according to a minimum object-size criterion. The image was then resized while preserving its aspect ratio, normalized using backbone-compatible mean and standard deviation, converted to the required channel format, and padded according to the size divisor required by the FPN structure. During validation and testing, the same deterministic preprocessing pipeline was used, except that random operations were removed.

All comparison models used the same lightweight augmentation strategy during training, consisting mainly of aspect-ratio-preserving resizing and random horizontal flipping with a probability of 0.5. Stronger augmentations, such as Mosaic, MixUp, aggressive random cropping, and heavy geometric transformations, were not used. FLS imagery is governed by acoustic imaging characteristics, and excessive augmentation may introduce unrealistic target-shadow relationships or distort the physical consistency of sonar echoes.

### 4.3. Quantitative Results

Table 1 and Table 2 summarize the comparison results on the two UATD test subsets. The proposed method achieves the highest overall detection accuracy among the compared detectors while maintaining a compact model size and low computational cost. It obtains 95.5% mAP@50 on UATD-Test-1 and 97.5% mAP@50 on UATD-Test-2. Compared with Hyper-YOLO-N, the strongest baseline in this comparison, the proposed method improves mAP@50 by 8.8 and 10.7 percentage points on UATD-Test-1 and UATD-Test-2, respectively.

These results indicate that applying general-purpose detectors to FLS imagery remains challenging without sufficient adaptation to acoustic imaging characteristics. In cluttered sonar scenes, background reverberation can be mistaken for valid targets, while weak target responses may lead to missed detections. Although transformer-based and two-stage detectors provide strong representation capacity, they often require higher computational cost or larger-scale labeled data. By adapting the architecture, loss design, and distillation strategy to FLS characteristics, the proposed framework achieves more stable performance across the two official UATD test subsets.

The lightweight student detector is still less competitive on the cube category in UATD-Test-1, where Sparse R-CNN and TOOD obtain slightly higher AP values. This may be related to the limited spatial detail preserved by the compact architecture when handling weak and ambiguous cube-like acoustic signatures. Nevertheless, the overall results show that the proposed method maintains a favorable accuracy–efficiency trade-off. The comparison with RF-DETR further suggests that general-purpose detectors may require additional adaptation before being applied to FLS imagery.

Table 3 reports the compact-target detection results on the two UATD test subsets. The proposed method obtains 59.19% AP_compact_ on UATD-Test-1 and 57.53% AP_compact_ on UATD-Test-2, giving the highest values among the compared methods under the same compact-target definition. These results indicate that the proposed framework remains effective for targets occupying only a small fraction of the FLS image.

The compact-target results should be interpreted together with the design of TADW. TADW is not a scale-aware loss and does not directly assign weights according to object size. Its benefit for compact targets comes from difficulty-aware supervision. In FLS imagery, compact targets are often associated with weak echoes, ambiguous boundaries, and low-confidence predictions, which are emphasized by the proposed reweighting mechanism. Combined with contextual aggregation in FPN-Mix and multi-level knowledge distillation, this supervision helps improve the detection of compact acoustic targets in cluttered sonar scenes.

The desktop-side complexity and inference-speed comparison is summarized in Table 4.

### 4.4. Visualization Results

**Figure 7:**
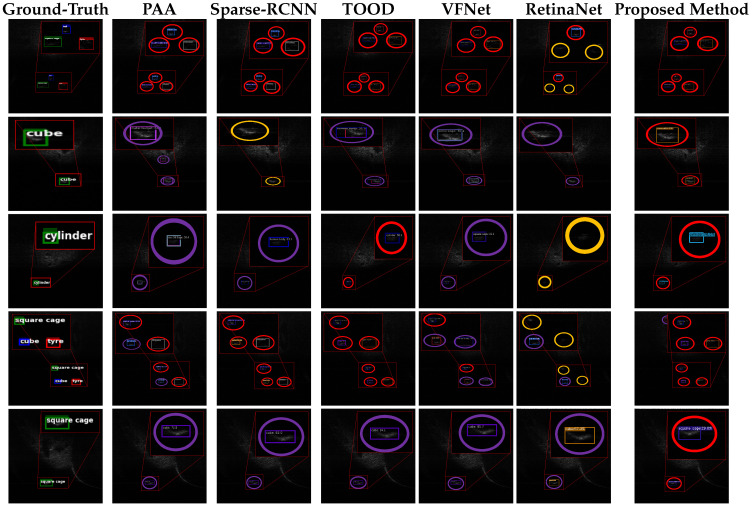
Qualitative comparison of detection performance achieved by different methods on the UATD-Test-1 dataset. Red, orange, and purple circles denote true positives, missed detections, and false alarms, respectively.

**Figure 8:**
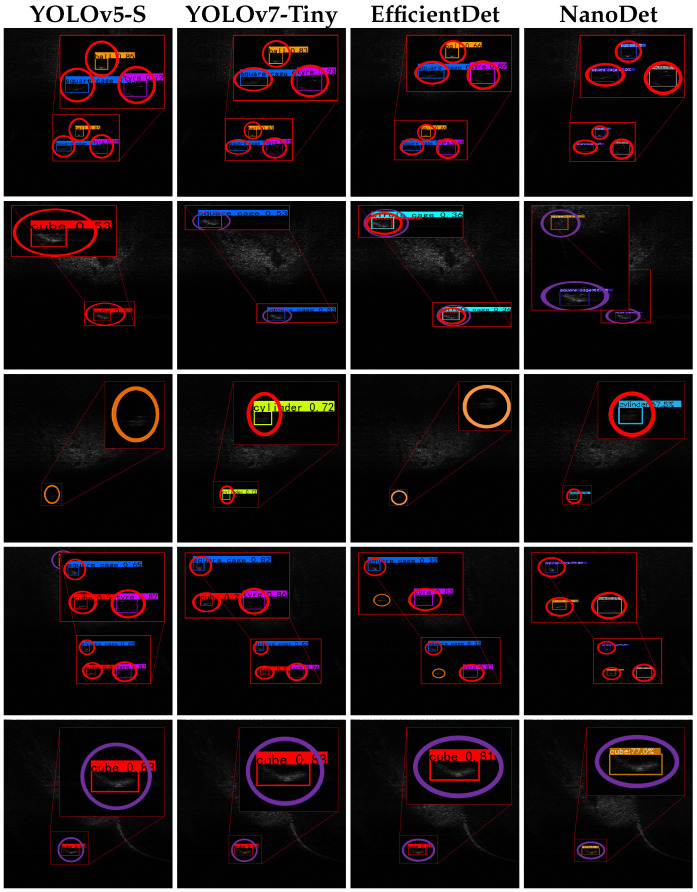
Qualitative detection results obtained by different methods on the UATD-Test-1 dataset. Red, orange, and purple circles denote true positives, missed detections, and false alarms, respectively.

Figure 7 and Figure 8 show representative detection results on the UATD dataset. Red, purple, and orange circles denote true positives, false alarms, and missed detections, respectively. Compared with SSS imagery, the FLS images in this dataset have lower contrast, weaker structural details, and stronger background interference, which makes target discrimination more difficult.

The visual results show that mainstream optical-image-oriented detectors still have limitations on FLS data. RetinaNet and Sparse R-CNN tend to miss complex underwater targets such as ROVs under cluttered conditions. TOOD can localize common sonar targets reasonably well, but it remains less reliable for weak-feature targets such as cylindrical structures. PAA also produces false alarms in background regions. These errors mainly arise from the mismatch between feature extraction mechanisms designed for optical images and the characteristics of FLS data, including multipath reflections, speckle noise, and weak low-contrast boundaries.

**Figure 9:**
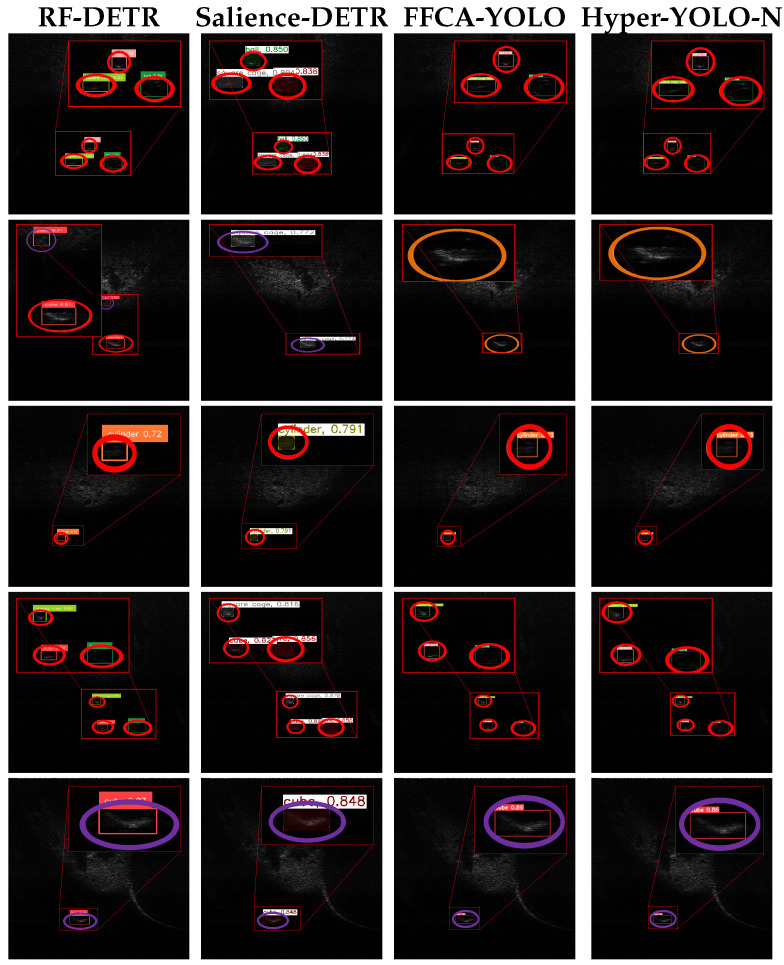
Qualitative detection results obtained by representative SOTA methods on the UATD-Test-1 dataset. Red, orange, and purple circles denote true positives, missed detections, and false alarms, respectively.

As shown in Figure 9, the representative SOTA detectors detect many targets under nominal conditions, but they still produce errors on harder classes and cluttered backgrounds. This behavior reflects the domain shift between optical imagery and FLS imagery, where intensity-only backscatter, low SNR, and geometry-dependent highlights and shadows weaken shape cues learned from optical data. In comparison, the proposed distillation framework provides more stable detection results by transferring discriminative knowledge from the teacher model to the lightweight student detector.

### 4.5. Computational Complexity Analysis

Table 4 reports the model complexity and desktop-side inference speed of different methods under a unified evaluation environment. The proposed method achieves a favorable trade-off between parameter count, computational cost, and detection accuracy.

### 4.6. Ablation Experiments

#### Component-WiseAblation Study

It is worth noting that the baseline loss used in this ablation study is the standard focal loss. Therefore, the comparisons in Table 5 and Table 6 directly reflect the effect of replacing focal loss with the proposed TADW loss under the same detector framework.

The ablation results on UATD-Test-1 and UATD-Test-2 are reported in Table 5 and Table 6, respectively. The overall trend is consistent across both test sets and aligns with the characteristics of FLS imagery, where weak target echoes, blurred structural boundaries, and severe reverberation interference make optimization particularly challenging for lightweight detectors.

Compared with the baseline detector, introducing TADW consistently improves the learning emphasis on difficult samples and alleviates the tendency of the model to be dominated by easy background responses. As a result, Baseline + TADW improves mAP@50 from 77.3% to 84.3% on UATD-Test-1 and from 77.6% to 83.2% on UATD-Test-2, confirming that adaptive sample reweighting is effective for suppressing background interference and enhancing weak-target discrimination in FLS imagery.

KD provides an additional gain by transferring more discriminative semantic and localization knowledge from the teacher model to the lightweight student. This is particularly beneficial in FLS imagery, where target appearance is often unstable and easily affected by clutter and acoustic scattering. Accordingly, Baseline + KD achieves 92.3%/59.09% mAP@50/mAP@50:95 on UATD-Test-1 and 94.4%/60.09% on UATD-Test-2, indicating that teacher-guided supervision can substantially strengthen the representation capacity of the lightweight detector.

When both modules are enabled, the Full Model achieves the best overall performance, reaching 95.5% mAP@50 and 60.0% mAP@50:95 on UATD-Test-1, and 97.5% mAP@50 and 61.1% mAP@50:95 on UATD-Test-2. These results indicate that TADW and KD are complementary for lightweight FLS target detection, jointly improving robustness to weak targets, clutter interference, and ambiguous sonar structures.

For the main comparison experiments, α was fixed at 1.5 to maintain a consistent setting across different detectors. To examine the sensitivity of TADW to this hyperparameter, additional experiments were conducted with α∈{2,2.5,3,3.5,4}. As shown in Table 7 and Table 8, the detection performance varies with α, but the trend is not strictly monotonic.

Compared with the default setting of α=1.5, moderate values around α=3 generally produce more favorable results on both test subsets. This suggests that an appropriate reweighting strength can help emphasize difficult acoustic samples without excessively disturbing the optimization process. When α is set too high, the performance shows a mild decline, indicating that overly strong reweighting may amplify hard or noisy samples and reduce training stability in cluttered FLS scenes.

### 4.7. Embedded Inference Benchmark on Jetson Orin NX

To evaluate the embedded inference capability of the proposed method, the trained model was exported to ONNX and benchmarked on an NVIDIA Jetson Orin NX platform. TensorRT FP16 acceleration was enabled during deployment, and the available execution providers were TensorRTExecutionProvider, CUDAExecutionProvider, and CPUExecutionProvider. The hardware and software configuration is summarized in Table 9.

For the ONNX/TensorRT deployment test, the model input size was read from the exported ONNX model and set to 416×416 in the benchmark. Each input image was resized while preserving its aspect ratio and padded to match the model input size. The image tensor was then converted to the required channel format and numerical precision before inference. The post-processing stage included confidence-based filtering, non-maximum suppression, and coordinate rescaling to map the predicted bounding boxes back to the original image size.

For runtime evaluation, 100 input images were used. Before formal measurement, a warm-up stage of 20 iterations was performed to reduce startup-related fluctuations. Both pure inference performance and end-to-end performance are reported. The pure inference benchmark reflects the execution efficiency of the deployed model itself, whereas the end-to-end benchmark additionally includes preprocessing and other runtime overhead, thereby providing a more realistic estimate of practical onboard deployment.

The benchmark results show that the proposed model achieves an average pure inference latency of 7.736 ms, corresponding to 129.26 FPS. When preprocessing and runtime overhead are included, the average end-to-end latency is 13.845 ms, corresponding to 72.23 FPS. These results indicate that the proposed lightweight framework maintains stable real-time performance on the Jetson Orin NX platform and is suitable for embedded FLS sensing scenarios under resource-constrained conditions.

**Figure 10:**
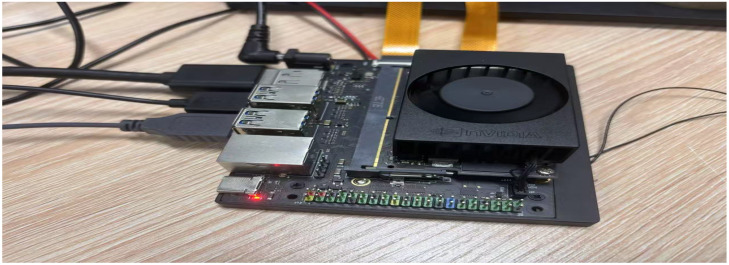
Embedded runtime validation setup of the proposed method on the NVIDIA Jetson Orin NX platform.

It should be noted that this experiment is intended to evaluate the on-device inference efficiency of the proposed method, rather than to provide a fully controlled cross-platform comparison against all baseline detectors. The desktop-side complexity and inference-speed comparison of different methods is reported separately in Table 4 under the same evaluation setting. The Jetson Orin NX benchmark is therefore used as an embedded deployment validation of the proposed model after ONNX export and TensorRT FP16 acceleration. The embedded runtime validation setup on the Jetson Orin NX platform is shown in Figure 10. The on-device resource usage and end-to-end runtime statistics are summarized in Table 10.

### 4.8. Generalization Evaluation on the Zhanjiang Bay No.1 Dataset

**Figure 11:**
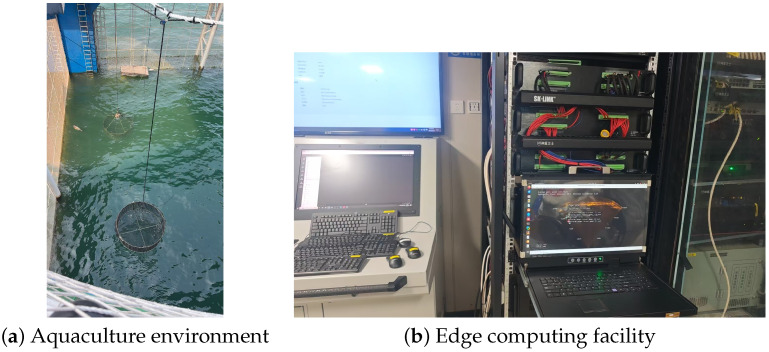
Deployment environment of the Zhanjiang Bay No.1 platform. (**a**) Aquaculture environment where the FLS data were collected. (**b**) Edge computing facility used for embedded deployment, which receives the sonar video stream, performs target detection, and pushes the processed video stream for aquaculture monitoring.

An additional FLS dataset collected from the Zhanjiang Bay No.1 aquaculture platform is used to evaluate field generalization in a practical marine aquaculture environment. The dataset was constructed from long-duration sonar video streams and differs from the public UATD benchmark in sensing conditions, target distribution, background structure, and deployment setting. The aquaculture environment and the edge computing facility are shown in Figure 11.

The sonar data were acquired using a Hanjie C750D forward-looking sonar (Shanghai Hanjie Technology Development Co., Ltd., Shanghai, China). operating in high-frequency mode with a sensing range of 20 m. Under this configuration, fish targets usually occupy a small region in the sonar image because the imaging range is large relative to the physical size of individual fish. This makes the dataset challenging for small-target detection. Fish in the Zhanjiang Bay No.1 platform also tend to stay close to the bottom region, producing bottom-concentrated target distributions. Cage structures, sediment background, acoustic reverberation, and clutter-like echoes further increase the difficulty of reliable detection.

The data were collected through an NVR-based continuous video recording pipeline. The FLS stream was transmitted through the aquaculture platform monitoring network to a network video recorder (NVR), where the incoming sonar stream was encoded, timestamped, and stored as long-duration video files. Each source video typically lasted several hours, preserving the temporal continuity of underwater sensing and the natural variation in fish distribution, platform background, and acoustic interference. Compared with manually selected still images, this recording process better reflects the data encountered in long-term aquaculture monitoring.

After data collection, the stored NVR videos were decoded frame by frame to obtain individual sonar images. Invalid frames, including frames with severe signal loss, transmission artifacts, or incomplete imaging regions, were removed during data screening. The final dataset contains approximately 20,000 valid sonar images. The dataset was divided into training, validation, and test subsets at a ratio of 6:2:2. Since the samples were extracted from continuous long-duration videos, the split was performed with temporal continuity in mind. Consecutive frames from the same temporal segment were assigned to the same subset rather than being randomly distributed across the training, validation, and test subsets. This protocol reduces the risk of data leakage caused by highly adjacent frames and makes the test set more suitable for evaluating model generalization under long-term monitoring conditions.

The deployment setting was also considered in this evaluation. As shown in Figure 11b, the proposed algorithm has been deployed on the edge computing box of the Zhanjiang Bay No.1 platform. During operation, the edge device receives the sonar video stream, performs target detection, and pushes the processed video stream for remote monitoring. This setting matches the goal of this work: a lightweight FLS detection framework that can operate under complex acoustic conditions and embedded computing constraints.

The proposed method is compared with four representative recent detectors on the Zhanjiang Bay No.1 dataset, including RF-DETR, Salience-DETR, FFCA-YOLO, and Hyper-YOLO. As reported in Table 11, the proposed method achieves 0.3940 mAP@50:95 and 0.7861 mAP@50, which are the highest values among the compared methods. The margin over Hyper-YOLO is limited, with gains of 0.0019 in mAP@50:95 and 0.0031 in mAP@50. Therefore, the result is better viewed as evidence of competitive field generalization rather than a substantial performance gap. Because the evaluation is conducted on an independently constructed field dataset containing approximately 20,000 sonar images from long-duration aquaculture monitoring videos, the result indicates that the proposed framework is not only fitted to the UATD benchmark but also remains effective under a different FLS data distribution.

**Figure 12:**
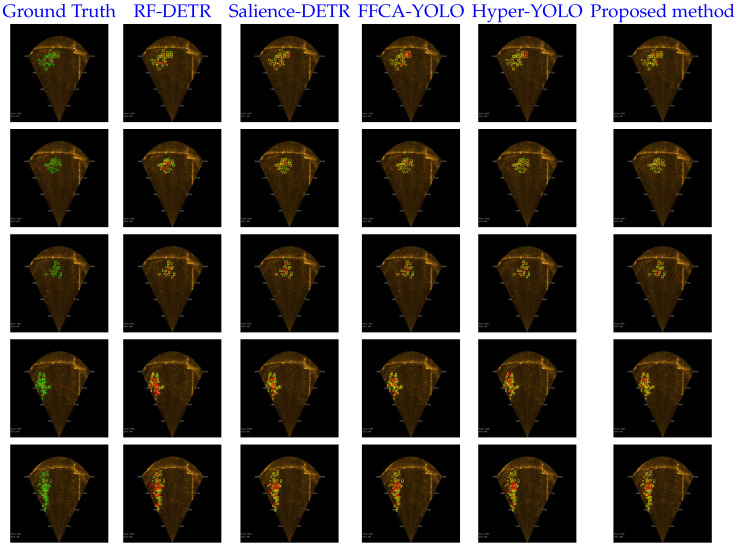
Qualitative comparison of detection results on the Zhanjiang Bay No.1 dataset. The green boxes indicate the ground-truth annotations, the yellow boxes indicate the predicted bounding boxes, and the red boxes mark representative missed-detection regions.

Figure 12 presents qualitative detection results on representative samples from the Zhanjiang Bay No.1 dataset. In comparison with the representative detectors, the proposed method detects more fish targets with fewer missed detections, especially in scenes characterized by bottom-concentrated fish distributions, weak acoustic responses, and cluttered aquaculture backgrounds. This indicates that the proposed framework improves target recall while maintaining more stable localization behavior under challenging field conditions. The qualitative results are consistent with the quantitative comparison reported in Table 11, providing further evidence of the effectiveness and practical applicability of the proposed method in real aquaculture scenarios.

## 5. Conclusions

This paper presented a lightweight forward-looking sonar (FLS) sensing framework for embedded target detection in resource-constrained underwater systems. The framework integrates an FPN-Mix backbone with the Conv-Mix module for contextual representation, a target-aware dynamic weighting (TADW) loss for difficult acoustic samples, and a multi-level knowledge distillation strategy for transferring feature-level and prediction-level knowledge from a stronger teacher model to the lightweight student detector.

Experiments on the public UATD benchmark demonstrate that the proposed method achieves a favorable accuracy–efficiency trade-off on both official test subsets. The model obtains 95.5% mAP@50 on UATD-Test-1 and 97.5% mAP@50 on UATD-Test-2, while requiring only 2.83M parameters and 6.68 GFLOPs. After ONNX export and TensorRT FP16 acceleration, the deployed model achieves 72.23 FPS in end-to-end inference on an NVIDIA Jetson Orin NX platform, supporting its feasibility for real-time embedded FLS deployment. Additional evaluation on the Zhanjiang Bay No.1 field dataset further indicates that the framework retains competitive generalization ability under a practical aquaculture sonar scenario.

Overall, the proposed framework provides a compact and deployable solution for FLS target detection under resource-constrained conditions. Nevertheless, although both public benchmark data and real-world platform data are evaluated, the current validation is still limited to specific sonar devices, operating ranges, and application scenarios. Future work will further investigate robustness under more diverse sonar configurations, seabed conditions, water environments, and long-term dynamic field deployments, and will extend the framework toward fish-group monitoring and continuous aquaculture sonar perception.

## Figures and Tables

**Table 1:** Performance comparison of the proposed method and representative detectors on the UATD-Test-1 dataset. The bold row indicates the proposed method.

Method	Cube	Ball	Cylin.	Human Body	Plane	Circle Cage	Square Cage	Metal Bucket	Tyre	ROV	mAP@50	mAP@50:95
RetinaNet	56.0%	79.0%	82.0%	82.0%	86.0%	47.0%	30.0%	75.0%	81.0%	65.0%	68.3%	26.8%
PAA	59.9%	80.4%	89.9%	76.5%	90.7%	54.4%	58.0%	74.2%	79.4%	93.0%	75.6%	32.5%
VFNet	55.3%	80.7%	65.2%	85.3%	90.9%	45.4%	49.1%	88.7%	71.8%	86.2%	71.9%	23.9%
Sparse												
R-CNN	69.1%	82.6%	96.0%	68.4%	90.7%	43.2%	61.1%	97.7%	73.9%	90.6%	77.3%	27.1%
TOOD	66.9%	78.0%	94.5%	86.8%	90.0%	58.1%	58.8%	100%	83.0%	90.4%	80.7%	26.9%
YOLOv5-S	67.97%	87.16%	74.47%	82.83%	95.32%	45.97%	55.52%	61.98%	79.02%	79.23%	72.9%	27.3%
YOLOv7-												
Tiny	60.19%	78.25%	98.48%	76.80%	98.19%	41.79%	55.91%	100%	90.12%	98.23%	79.8%	30.1%
EfficientDet-												
Lite-D1	52.18%	79.72%	88.97%	77.36%	97.20%	54.36%	47.51%	63.3%	83.62%	70.6%	71.5%	26.3%
NanoDet	59.3%	85.4%	95.8%	83.6%	97.8%	55.0%	58.6%	100%	88.6%	97.5%	82.2%	29.3%
RF-DETR	69.3%	79.36%	98.72%	81.15%	98.73%	43.89%	61.1%	77.2%	81.81%	98.76%	79.0%	37.83%
Salience-												
DETR	64.3%	85.4%	88.0%	83.6%	96.5%	62.4%	60.7%	83.2%	76.9%	91.3%	79.2%	34.9%
FFCA-												
YOLO	62.2%	85.9%	96.8%	77.0%	98.2%	48.5%	61.5%	53.7%	82.3%	94.1%	76.0%	31.3%
Hyper-												
YOLO-N	83.6%	86.3%	98.1%	88.6%	98.2%	65.9%	64.1%	99.5%	87.6%	94.9%	86.7%	39.3%
**Proposed Method**	**66.7%**	**99.8%**	**97.6%**	**98.4%**	**98.7%**	**99.4%**	**99.5%**	**98.6%**	**96.5%**	**100%**	**95.5%**	**60.0%**

**Table 2:** Performance comparison of the proposed method and representative detectors on the UATD-Test-2 dataset. The bold row indicates the proposed method.

Method	Cube	Ball	Cylin.	Human Body	Plane	Circle Cage	Square Cage	Metal Bucket	Tyre	ROV	mAP@50	mAP@50:95
RetinaNet	58.0%	78.0%	79.0%	87.0%	89.0%	47.0%	29.0%	88.0%	87.0%	52.0%	69.4%	27.5%
PAA	60.2%	80.9%	86.8%	85.2%	93.5%	54.8%	50.7%	100%	84.0%	91.1%	78.7%	27.6%
VFNet	51.1%	81.9%	60.3%	85.9%	90.7%	46.5%	50.0%	42.9%	71.8%	83.6%	66.5%	23.2%
Sparse												
R-CNN	65.8%	81.5%	87.1%	88.4%	90.9%	46.2%	53.0%	100%	82.2%	90.6%	78.6%	26.9%
TOOD	65.0%	85.4%	88.4%	87.5%	99.5%	67.6%	49.5%	100%	86.7%	89.7%	81.9%	30.2%
YOLOv5-S	62.88%	87.77%	84.12%	82.62%	97.83%	50.75%	50.69%	89.09%	82.58%	78.5%	76.7%	27.8%
YOLOv7-												
Tiny	61.34%	74.70%	94.86%	91.71%	99.57%	50.75%	55.96%	100%	89.08%	95.74%	81.4%	32.3%
EfficientDet-												
Lite-D1	47.87%	79.33%	81.69%	89.11%	98.78%	54.91%	45.15%	47.08%	84.45%	64.10%	69.2%	21.7%
NanoDet	59.8%	82.5%	96.8%	91.9%	100%	59.9%	53.2%	100%	92.5%	98.7%	83.5%	31.3%
RF-DETR	61.3%	83.70%	81.93%	89.19%	97.42%	45.58%	60.35%	100%	86.15%	99.04%	80.5%	35.61%
Salience-												
DETR	60.4%	88.7%	83.0%	83.7%	99.6%	68.4%	59.5%	72.3%	83.9%	87.8%	78.7%	34.5%
FFCA-												
YOLO	64.1%	83.9%	94.6%	89.7%	99.4%	47.4%	57.8%	99.5%	84.9%	92.6%	81.4%	33.1%
Hyper-												
YOLO-N	82.3%	86.9%	94.1%	95.5%	99.2%	66.8%	59.0%	99.5%	93.1%	91.1%	86.8%	40.0%
**Proposed Method**	**90.3%**	**98.3%**	**99.2%**	**98.7%**	**94.5%**	**96.4%**	**98.6%**	**99.5%**	**99.7%**	**100%**	**97.5%**	**61.1%**

**Table 3:** Compact-target detection performance on the UATD test subsets. Compact targets are defined as targets with a relative bounding-box area smaller than 0.3% of the original image area.

Method	${\mathrm{AP}}_{\mathrm{compact}}$ on UATD-Test-1	${\mathrm{AP}}_{\mathrm{compact}}$ on UATD-Test-2
PAA	50.15%	55.50%
VFNet	43.86%	42.17%
TOOD	49.68%	52.76%
Sparse R-CNN	51.54%	55.12%
NanoDet	57.67%	56.70%
RetinaNet	47.68%	44.78%
YOLOv7-Tiny	49.96%	50.03%
YOLOv5-S	49.30%	55.97%
Salience-DETR	57.19%	55.50%
Hyper-YOLO-N	56.20%	56.40%
RF-DETR	51.02%	54.67%
Proposed Method	59.19%	57.53%

**Table 4:** Desktop-side complexity and inference speed comparison of different methods under the same evaluation setting. The bold row indicates the proposed method.

Method	Params (M)	GFLOPs	FPS
Teacher Model	82.68	323.16	38.74
RetinaNet	36.517	148.159	19.9
PAA	32.134	51.6178	13.6
VFNet	32.73	48.43	21.0
TOOD	32.3	51.449	24.2
Sparse R-CNN	106	32.925	19.2
YOLOv5-S	7.2	16.95	27.73
YOLOv7-Tiny	6.2	13.8	41.26
EfficientDet-Lite-D1	6.6	6.1	58.53
NanoDet	**1.147**	**0.738**	64.20
RF-DETR	27.16	38.96	20.26
Salience-DETR	227	85.5	5.77
FFCA-YOLO	7.13	51.3	168
Hyper-YOLO-N	3.62	9.5	176
**Proposed Method**	**2.83**	**6.68**	**155**

**Table 5:** Ablation comparison of different TADW/KD combinations on the UATD-Test-1 dataset. Here, ✓ and × indicate that the corresponding component is enabled and disabled, respectively. The bold row denotes the full-model configuration.

Model Variant	TADW	KD	Cube	Ball	Cylinder	Human Body	Plane	Circle Cage	Square Cage	Metal Bucket	Tyre	ROV	${\mathrm{mAP}}_{50}$	${\mathrm{mAP}}_{50:95}$
Baseline	×	×	24.7%	92.3%	74.5%	85.4%	89.0%	66.2%	77.8%	82.3%	91.1%	89.9%	77.3%	43.2%
Baseline + TADW	✓	×	38.6%	91.0%	87.6%	88.7%	89.7%	90.5%	90.9%	91.6%	82.5%	91.7%	84.3%	44.4%
Baseline + KD	×	✓	60.0%	100%	96.5%	97.4%	96.4%	87.0%	95.6%	93.4%	100%	97.1%	92.3%	59.09%
**Full Model**	✓	✓	**66.7%**	**99.8%**	**97.6%**	**98.4%**	**98.7%**	**99.4%**	**99.5%**	**98.6%**	**96.5%**	**100.0%**	**95.5%**	**60.0%**

**Table 6:** Ablation comparison of different TADW/KD combinations on the UATD-Test-2 dataset. The bold row denotes the full-model configuration.

Model Variant	TADW	KD	Cube	Ball	Cylin.	Human Body	Plane	Circle Cage	Square Cage	Metal Bucket	Tyre	ROV	${\mathrm{mAP}}_{50}$	${\mathrm{mAP}}_{50:95}$
Baseline	×	×	29.3%	90.5%	75.3%	86.0%	88.6%	67.0%	78.0%	82.7%	88.7%	89.5%	77.6%	46.6%
Baseline + TADW	✓	×	38.9%	89.5%	87.3%	87.7%	88.0%	88.8%	89.3%	89.9%	81.9%	90.3%	83.2%	49.4%
Baseline + KD	×	✓	71.4%	100%	98.5%	97.5%	96.4%	89.0%	97.3%	93.4%	100%	100%	94.4%	60.09%
**Full Model**	✓	✓	**90.3%**	**98.3%**	**99.2%**	**98.7%**	**94.5%**	**96.4%**	**98.6%**	**99.5%**	**99.7%**	**100.0%**	**97.5%**	**61.1%**

**Table 7:** Sensitivity analysis of different α values in TADW on UATD-Test-1. The bold value indicates the best result.

α	Cube	Ball	Cylinder	Human Body	Plane	Circle Cage	Square Cage	Metal Bucket	Tyre	ROV	${\mathrm{mAP}}_{50}$	${\mathrm{mAP}}_{50:95}$
α=2	65.8%	99.7%	97.2%	98.2%	98.4%	99.2%	99.3%	98.3%	95.9%	99.0%	95.1%	59.8%
α=2.5	66.1%	99.8%	97.4%	98.4%	98.5%	99.3%	99.4%	98.5%	96.1%	99.0%	95.3%	60.7%
α=3	**70.2%**	**99.8%**	**98.3%**	**99.0%**	**99.0%**	**99.6%**	**99.6%**	**99.0%**	**97.5%**	**100.0%**	**96.2%**	**61.6%**
α=3.5	68.9%	99.8%	98.0%	98.7%	98.8%	99.5%	99.5%	98.8%	97.0%	100.0%	95.9%	61.2%
α=4	65.6%	99.5%	97.1%	98.1%	98.1%	99.1%	99.1%	98.2%	95.2%	100.0%	95.0%	59.9%

**Table 8:** Sensitivity analysis of different α values in TADW on UATD-Test-2. The bold value indicates the best result.

α	Cube	Ball	Cylin.	Human Body	Plane	Circle Cage	Square Cage	Metal Bucket	Tyre	ROV	${\mathrm{mAP}}_{50}$	${\mathrm{mAP}}_{50:95}$
α=2	91.4%	98.3%	97.9%	97.7%	95.5%	96.2%	93.6%	94.3%	99.7%	100.0%	95.6%	60.1%
α=2.5	93.2%	98.3%	99.3%	98.9%	94.6%	96.4%	94.7%	99.1%	99.7%	99.4%	97.4%	62.7%
α=3	**95.5%**	**98.3%**	**99.5%**	**99.4%**	**97.5%**	**97.2%**	**98.6%**	**99.5%**	**99.7%**	**99.5%**	**98.5%**	**63.8%**
α=3.5	93.7%	98.3%	99.2%	99.4%	95.5%	96.8%	97.7%	99.5%	99.7%	98.4%	97.8%	63.7%
α=4	94.1%	98.3%	97.6%	96.3%	95.5%	96.0%	97.8%	98.6%	94.4%	97.6%	96.6%	61.0%

**Table 9:** Hardware and software configuration of the Jetson Orin NX embedded inference platform.

Item	Configuration
**Device**	NVIDIA Jetson Orin NX embedded platform
**CPU**	8-core ARMv8 processor
**GPU**	NVIDIA Jetson Orin NX integrated GPU
**Memory**	15.3 GiB
**Storage**	128 GB
**Operating System**	Ubuntu 22.04.5 LTS (64-bit)
**Deployment Engine**	ONNX + TensorRT FP16

**Table 10:** On-device resource usage and end-to-end runtime statistics of the proposed method on the Jetson Orin NX platform.

Method	Power (mW)	GPU Memory (MB)	Latency (ms)	End-to-End FPS
Proposed Method	5951	83	13.845	72.23

**Table 11:** Comparison with representative recent detectors on the Zhanjiang Bay No.1 dataset.

Method	mAP@50:95	mAP@50
RF-DETR	0.3687	0.7599
Salience-DETR	0.3778	0.7691
FFCA-YOLO	0.3864	0.7783
Hyper-YOLO	0.3921	0.7830
Proposed Method	**0.3940**	**0.7861**

## Data Availability

The data used in this study are available from the corresponding author upon reasonable request.
